# Molecular Biology: Navigating the Landscape

**DOI:** 10.3390/cimb48020133

**Published:** 2026-01-26

**Authors:** Madhav Bhatia

**Affiliations:** Department of Pathology and Molecular Medicine, University of Otago, Christchurch, 2 Riccarton Avenue, P.O. Box 4345, Christchurch 8140, New Zealand; madhav.bhatia@otago.ac.nz or madhav1245@gmail.com

“Latest Review Papers in Molecular Biology 2025”, a Special Issue of *Current Issues in Molecular Biology (CIMB)*, was open for publishing review articles on navigating the landscape in molecular biology. It has been a privilege editing this Special Issue. The call for papers for this Special Issue received an excellent response from researchers around the world, and this Special Issue has now published 31 articles (26 January 2026), which have all helped to enhance our understanding of the current issues in the discipline of molecular biology. The articles published in this Special Issue have already been viewed 86,458 times at the time of writing (13 January 2026), and this number is rising.

The articles in this Special Issue are of an extremely high standard, and it has been my pleasure to read and edit them. I would encourage the readers of *CIMB* to read all the articles in this Special Issue, if possible. I am sure you will enjoy reading them, just like I have. I would, however, like to highlight a few noteworthy review articles published in the Special Issue. In this Editorial, I have chosen to highlight the five articles that have been viewed the greatest number of times to date.

The first article [1] describes the therapeutic potential of alpha lipoic acid (ALA) and its role in oxidative stress and inflammatory conditions. ALA is an essential organosulfur compound with varied therapeutic applications. The review article describes our current understanding of the multifaceted role of ALA in several inflammatory diseases (acute pancreatitis, arthritis, osteoarthritis, asthma, and sepsis), cardiovascular disorders, and neurological conditions. The article also describes evidence from the literature showing that ALA modulates the signaling pathways involved in inflammation and cellular oxidative stress responses, making it a promising candidate for mitigating inflammation. Furthermore, the article offers a novel perspective that attributes some of the therapeutic effects of ALA to its ability to release hydrogen sulfide, a gaseous signaling molecule [2,3]. In light of the interest in this rapidly developing area, in the short time since it was published, the article has been viewed 13,701 times and cited 6 times.

The second article [4] describes methane, bacteria, fungi, and fermentation in the gut and discusses pathophysiology, diagnosis, and treatment strategies for small intestinal bacterial overgrowth, intestinal methanogen overgrowth, and small intestinal fungal overgrowth. Human gut microbiota play a key role in digestion and immune system function. Among the clinically recognized manifestations of dysbiosis in this system are small intestinal bacterial overgrowth, intestinal methanogen overgrowth, small intestinal fungal overgrowth, and large intestinal bacterial overgrowth [5]. The review aimed to investigate the complex pathophysiological mechanisms underlying these syndromes and their diagnostic and therapeutic options, focusing primarily on the roles of methane-producing archaea and fungal overgrowth. The article highlighted the complex and multifactorial nature of gut dysbiosis, pointing to its impact beyond the gastrointestinal tract. The article also suggested directions for future research in this area, given the current limitations of our understanding of the subject. To date, the article has been viewed 7496 times and cited 1 time.

The third article [6] describes the impact and molecular mechanisms of exercise in cancer therapy. Exercise plays an important supporting role in cancer treatment [7]. The article describes the contribution of physical exercise to cancer prevention and treatment, emphasizing its positive effects on reducing fatigue, improving physical strength, and enhancing mental health. It also summarizes our current understanding of the molecular mechanisms regulating tumor immunity and energy metabolism. Moreover, the article covers the criteria for selecting exercise types and intensities and the development of personalized exercise plans. Finally, it provides guidelines for exercise prescriptions and suggests future research directions for improving interventions for cancer patients. The article has been viewed 7469 times and cited 2 times so far.

The fourth article [8] describes recent advances in bone tissue engineering (BTE) and suggests ways of enhancing the potential of mesenchymal stem cells for use in regenerative therapies. BTE is promising strategy for addressing bone defects and disorders that cannot be repaired through traditional methods [9]. Mesenchymal stem cells (MSCs) have excellent osteogenic potential, which can be enhanced through osteoinductive factors. Bioprinting technologies have opened new avenues for precisely designing scaffolds that can mimic the native bone architecture and provide a conducive environment for MSC differentiation. Integrating bioprinting with MSCs and osteoinductive factors allows for creating patient-specific bone grafts. This review highlights the latest developments in MSC-based therapies, the role of osteoinductive factors, and the impact of bioprinting in advancing BTE. The article also describes the current state of the art and potential future directions for this very important and rapidly developing subject. The article has been viewed 6106 times and cited 7 times so far.

Finally, the fifth article summarizes our current understanding of the role of bone morphogenetic proteins (BMPs) and their biological mechanisms [10]. BMPs are cytokines that belong to the Transforming Growth Factor (TGF)-β family and perform diverse roles in development, osteogenesis, and vasculogenesis. The dysregulation of BMP activity at various stages in signal transduction is associated with a diverse range of human diseases [11]. The review describes the dysregulation of BMPs identified in various cancer types, which serves as a predictive sign for favorable results in cancer therapy. Alterations in certain components of the BMP pathway are evident in various types of cancer, including breast, gastric, colorectal, and myeloma cancer. The review reinforces the conclusion that BMPs can exert both beneficial and detrimental effects on cancer biology. The article points to a novel direction for treating different forms of cancer. Like the other articles that I have highlighted in this Editorial, this article deals with a complex subject and explains it in a very easy-to-read manner. So far, the article has been viewed 5300 times and cited 4 times.

In conclusion, this Special Issue represents a collection of review papers from varied scientific fields in the discipline of molecular biology. This Special Issue was a successor to “Latest Review Papers in Molecular Biology 2024”, which was also very popular with scholars working in the discipline of molecular biology. Encouraged by the response to the Special Issues in 2024 and 2025—from the authors as well as readers—we have set up another Special Issue of *CIMB*, “Latest Review Papers in Molecular Biology 2026” (https://www.mdpi.com/journal/cimb/special_issues/6VOYE10R60 accessed 13 January 2026), and I invite you to contribute review articles on navigating the landscape in molecular biology.
